# Ultraviolet-Visible and Fluorescence Spectroscopy Techniques Are Important Diagnostic Tools during the Progression of Atherosclerosis: Diet Zinc Supplementation Retarded or Delayed Atherosclerosis

**DOI:** 10.1155/2013/652604

**Published:** 2013-11-13

**Authors:** Mohamed Anwar K. Abdelhalim, Sherif A. Abdelmottaleb Moussa, Yanallah Hussain AL-Mohy

**Affiliations:** ^1^Department of Physics and Astronomy, College of Science, King Saud University, P.O. Box 2455, Riyadh 11451, Saudi Arabia; ^2^Department of Physics, College of Science, Al-Imam Muhammad Ibn Saud Islamic University, P.O. Box 90950, Riyadh 11623, Saudi Arabia; ^3^Biochemistry Department, Biophysics group, National Research Centre, Dokki, Giza, Egypt

## Abstract

*Background.* In this study, we examined whether UV-visible and fluorescence spectroscopy techniques detect the progression of atherosclerosis in serum of rabbits fed on high-cholesterol diet (HCD) and HCD supplemented with zinc (HCD + Zn) compared with the control. *Methods.* The control rabbits group was fed on 100 g/day of normal diet. The HCD group was fed on Purina Certified Rabbit Chow supplemented with 1.0% cholesterol plus 1.0% olive oil (100 g/day) for the same period. The HCD + Zn group was fed on normal Purina Certified Rabbit Chow plus 1.0% cholesterol and 1.0% olive oil supplemented with 470 ppm Zn for the same feeding period. UV-visible and fluorescence spectroscopy and biochemistry in Rabbit's blood serum and blood hematology were measured in Rabbit's blood. *Results.* We found that the fluorescent peak of HCD shifted toward UV-visible wavelength compared with the control using fluorescent excitation of serum at 192 nm. In addition, they showed that supplementation of zinc (350 ppm) restored the fluorescent peak closely to the control. By using UV-visible spectroscopy approach, we found that the peak absorbance of HCD (about 280 nm) was higher than that of control and that zinc supplementation seemed to decrease the absorbance. *Conclusions.* This study demonstrates that ultraviolet-visible and fluorescence spectroscopy techniques can be applied as noninvasive techniques on a sample blood serum for diagnosing or detecting the progression of atherosclerosis. The Zn supplementation to rabbits fed on HCD delays or retards the progression of atherosclerosis. Inducing anemia in rabbits fed on HCD delays the progression of atherosclerosis.

## 1. Background 

Atherosclerosis is the primary cause of coronary and cardiovascular diseases. Atherosclerosis and heart diseases are major causes of morbidity and mortality in adults in industrialized nations. Atherosclerosis, a stenotic lesion of arterial walls, is a leading cause of death affecting almost one third of humans in developed countries. It can generally be viewed as a form of chronic inflammation that is induced and perturbed by lipid accumulation [1, 2]. Hyperlipidemia or a high serum triacylglycerol and cholesterol level is considered an important risk factor for the progression of atherosclerosis. The causes of atherosclerosis are mainly due to lifestyle, cholesterol-rich diet, and hereditary [3]. 

Atherosclerotic lesions are characterized by progressive thickening of the arterial intima layer due to cellular proliferation (foam cells), lipid and cholesterol deposition. Following extracellular lipid accumulation, there is a change in elastin layer and enlargement of upper intima with development of a “collagenous cap” or fibroatheroma plaque. In advanced lesions lipid and necrotic deposits accumulate within the intima, and calcium crystals build-up takes place, leading to the ultimate calcification of artery wall. Although many therapies are available for treatment, usually the physician's intervention occurs when the plaque is symptomatic, with high commitment of important organs that need high blood supply, such as heart and brain [4–7]. 

Fluorescence spectroscopy has been recently explored as a new technique for the diagnosis of disease in human tissue [8–11]. Several groups have demonstrated that normal and atherosclerotic human artery can be differentiated on the basis of their fluorescence emission spectra. Many have suggested that this represents a new technique for determining the histochemical composition of atherosclerotic plaque *in vivo *and potentially guiding laser angiosurgery catheters [8–10]. Similarly, it has been shown that neoplastic tissue can be discriminated from nonneoplastic tissue, in a variety of organs, including the colon [10] and lung [12] on the basis of tissue fluorescence emission spectra. 

It has been reported that feeding rabbits a high-cholesterol diet resulted in alterations in prooxidant-antioxidant status in several tissues as well as typical atherosclerotic changes in the aorta [13–16].

Fluorescence is the emission of light by a substance following absorption of light or other electromagnetic radiation of a different wavelength. In most cases, the emitted light has a longer wavelength, and therefore lower energy, than the absorbed radiation. However, when the absorbed electromagnetic radiation is intense, it is possible for one molecule to absorb two photons; this two-photon absorption can lead to emission of radiation having a shorter wavelength than the absorbed radiation.

The Fourier-Transform Raman (FT-Raman) spectroscopy is an optical tool which permits less invasive and nondestructive analysis in biological samples with high precision, allowing one to get precision information of biochemical composition from different types of human tissues such as coronary arteries, lung, and colon without tissue removal [17–19]. The optical diagnosis has been used in a variety of human diseases, such as near-infrared fluorescence spectroscopy of Alzheimer disease, near-infrared Raman spectroscopy of cervical precancers, and human breast cancer analysis. 

Currently there is no noninvasive clinical method available for detecting or diagnosing atherosclerotic plaque content without withdrawing tissue. The ultraviolet-visible and fluorescence spectroscopy techniques have not been well documented in rabbits blood fed on high-cholesterol diet (HCD) and HCD supplemented with high zinc (Zn) level. Thus, the aim of this study is to diagnosis or detect the progression of atherosclerosis as well as the delay or retard of the progression of atherosclerosis applying ultraviolet-visible and fluorescence spectroscopy techniques on rabbit's blood sample. For this reason, to diagnose atherosclerosis with noninvasive techniques, a group of rabbits are fed on HCD for a feeding period of 12 weeks, and rabbits are fed on HCD supplemented with high Zn level for the same feeding period of time; then ultraviolet-visible and fluorescence spectroscopy techniques will be applied as noninvasive techniques on rabbit's blood sample.

## 2. Materials and Methods

### 2.1. Animals

The atherosclerotic model used in this study was the New Zealand white rabbit (male, 12 weeks old), obtained from the Laboratory Animal Center (College of Pharmacy, King Saud University). Fifteen rabbits were individually caged and divided into the following. The 1st group is control group (*n* = 8), the 2nd group is high-cholesterol diet (HCD; *n* = 8) group, and the 3rd group is the HCD group supplemented with high Zn level (*n* = 8). The control group (*n* = 5) was fed on 100 g/day of NOR diet (Purina Certified Rabbit Chow no. 5321; Research Diet Inc., New Jersey, USA) for a feeding period of 12 weeks. Chemical composition of the laboratory NOR rabbit diet (Purina Certified Rabbit Chow # 5321) [4–6]. The HCD group (*n* = 5) was fed on NOR Purina Certified Rabbit Chow no. 5321 supplemented with 1.0% cholesterol plus 1.0% olive oil (100 g/day) for the same feeding period of time. The HCD + Zn group was fed on NOR Purina Certified Rabbit Chow plus 1.0% cholesterol and 1.0% olive oil supplemented with 350 ppm Zn (total estimate 470 ppm Zn) for the same feeding period of time. The animals were sacrificed by intravenous injection of Hypnorm (0.3 mL/kg) in accordance with the guidelines approved by King Saud University Local Animal Care and Use Committee.

### 2.2. Collection of Blood Serum

Blood samples were collected from control, HCD, and HCD + Zn groups of animals, following an overnight fast, and collectively analyzed. Blood samples of 2 mL were obtained from the rabbits via vein puncture of an antecubital vein. The blood was collected into two polypropylene tubes, one for serum and one for plasma. The blood for plasma was collected in heparin. The serum was prepared by allowing the blood to clot at 37°C and centrifuge at 3000 rpm for 10 min.

### 2.3. UV-Visible Spectroscopy

#### 2.3.1. Absorbance Spectra

Rabbit blood serum absorbance of control, HCD, and HCD + Zn groups was measured using a UV-VIS double beam spectrophotometer (UV-1601 PC, Shimadzu, Japan; H14 grating UV) through optical resolution of 0.4 nm. Absorbance measurements were obtained over wavelength range of 200–800 nm at room temperature using quartz cuvettes (1 cm path length). The cuvettes were cleaned before each use by sonicating for 5 min in deionized water followed by rinsing with deionized water. The pH for different blood serum samples was kept constant during the measurements.

#### 2.3.2. Fluorescence Spectroscopy

Fluorescence measurements were performed in rabbit's blood serum samples using a FluoroMax-2 JOBAN YVON-SPEX, Instruments S.A., Inc., France. The fluorescence measurements were made over wavelength range of 190–400 nm and at 2 different excitation values (192 and 278 nm) on series of samples with a 10 mm light path cuvette.

### 2.4. Biochemical and Hematological Investigations

Blood samples (2 mL) were obtained from the rabbits via vein puncture of an antecubital vein. Blood was collected into two polypropylene tubes, namely, one for serum and one for plasma. The blood for plasma was collected in heparin. Serum was prepared by allowing the blood to clot at 37°C and centrifugation at 3000 rpm for ten minutes. A hematological autoanalyzer (Orphee Mythic 22 Hematological Analyzer, Diamond Diagnostic, USA) was used to determine different hematological and dimensional parameters, such as red blood cells (RBC), white blood cells (WBCs), hemoglobin (HB), hematocrit (HCT), mean corpuscular volume (MCV), mean corpuscular hemoglobin (MCH), mean corpuscular hemoglobin concentration (MCHC), red blood cell distribution width (RDW), neutrophils%, lymphocytes%, monocytes%, eosinophils%, basophils%, mean platelet volume (MPV), platelet distribution width (PDW)%, plateletcrit (PCT)%, and platelets (PLTs). Serum TC and TG levels were analyzed by the enzymatic method used in the clinical laboratory center of King Khaled Hospital. HDL concentration was determined by the previously reported method (Lee et al., 1998).

### 2.5. Histopathological Analysis

Animals were weighted and examined daily for health and behavioral changes following HCD and HCD + Zn administration. To evaluate the morphological changes, several samples from different rabbit organs (liver, heart, lung, kidney, and aorta) were cut rapidly, fixed in neutral buffered formalin (10%), and dehydrated through a series of ethanol grades (70, 80, 90, 95, and 100%). The rabbit samples were then cleared in 2 changes of xylene, impregnated with 2 changes of molten paraffin wax, embedded, and blocked. Paraffin sections (4-5 um) were stained with hematoxylin and eosin according to [20]. 

Traditional histopathological technique was performed. Hematoxylin and eosin, Verhoeff, Masson, and Picrosirius staining were used for tissue structure observation. The histopathological classification was made by a board certified pathologist. 

### 2.6. Statistical Analysis

We expressed the results for UV-Visible and fluorescence spectroscopy, blood biochemistry, and hematology as mean ± standard error (Mean ± SE). To assess the significance of the differences between control, HCD, and HCD + Zn, we performed the statistical analysis using one-way analysis of variance (ANOVA) for repeated measurements, with significance assessed at 5% confidence level.

## 3. Results and Discussions

To diagnose or detect and delay the progression of atherosclerosis, noninvasive techniques such as ultraviolet-visible and fluorescence spectroscopy techniques were applied on rabbit's blood sample fed on HCD and HCD supplemented with high Zn level for a feeding period of 12 weeks. 

### 3.1. UV-Visible Spectroscopy of Rabbit's Blood Serum

The normal blood is characterized by Five hemoglobin (Hb) peaks: the peak at 280 nm corresponds to the aromatic amino acids, at 340 nm corresponds to globin-heme interaction, at 420 nm corresponds to the heme, the peak at 540 nm corresponds to heme-heme interactions, and at 578 nm corresponds to hemoglobin oxygen affinity [7, 21]. 

**Figure 1:**
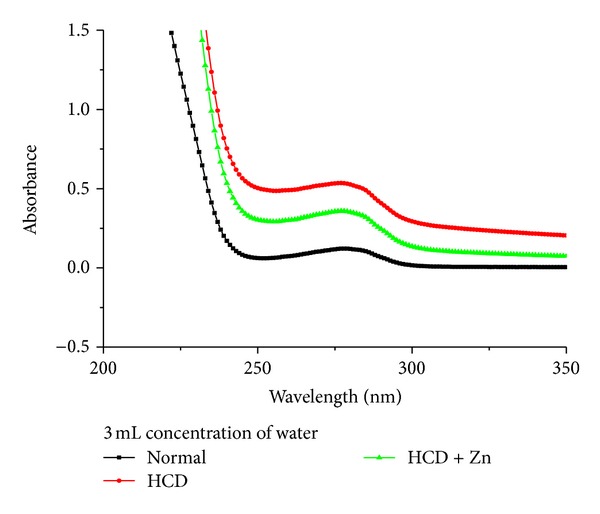
It shows absorbance against wavelength (nm) for a sample of rabbit's blood serum taken from control, HCD, and HCD + Zn groups (3 mL concentration of water).

**Figure 2:**
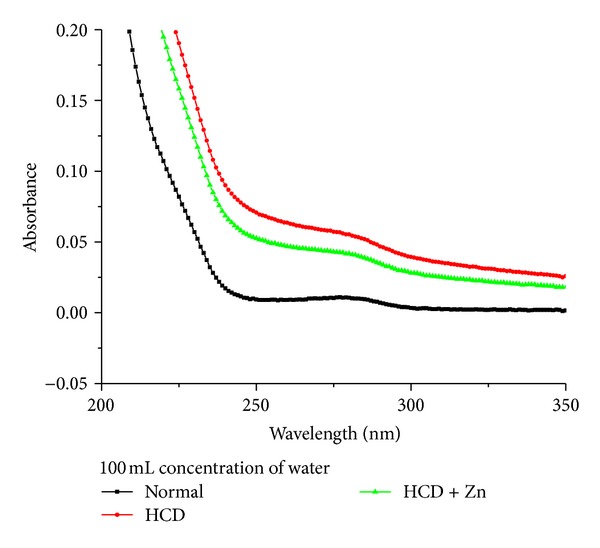
It shows absorbance against wavelength (nm) for a sample of rabbit's blood serum taken from control, HCD, and HCD + Zn groups (100 mL concentration of water).

Figures 1 and 2 show the average absorbance against wavelength (nm) for rabbit's blood serum taken from control, HCD, and HCD + Zn groups (*N* = 5). In each group, the absorbance peak for rabbits fed on HCD significantly increased compared with the control, while it returned towards the control absorbance peak for rabbits fed on HCD + Zn. The absorbance peak for control was significantly different from those for HCD and HCD + Zn.

### 3.2. Fluorescence Spectroscopy of Rabbit's Blood Serum

**Figure 3:**
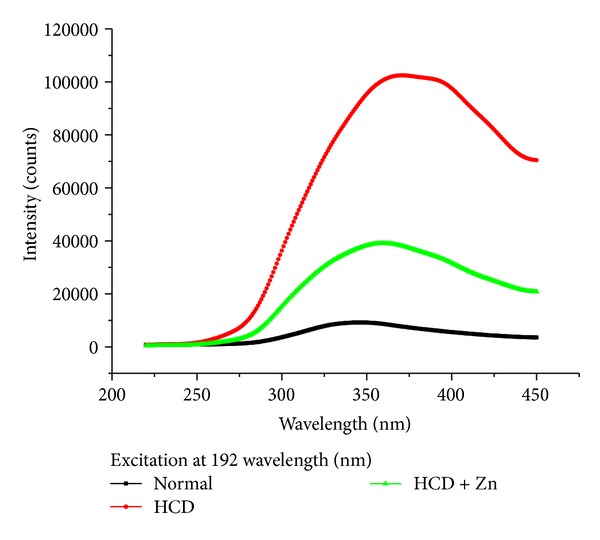
Fluorescence intensity (counts) against wavelength (nm) for a sample of rabbit's blood serum taken from control, HCD, and HCD + Zn groups at excitation wavelength of 192 nm.

We have observed two peaks at wavelengths 195 nm and 280 nm using the UV-Visible spectroscopy (Figures 1 and 2). Thus in the methodological design of this study we have done excitation at wavelengths 192 and 278 nm for fluorescence spectroscopic analysis of rabbit blood serum [7].  Figure 3 shows fluorescence intensity (counts) against wavelength (nm) for a sample of rabbit's blood serum taken from control, HCD, and HCD + Zn groups. In which an excitation wavelength of 192 nm was used to induce autofluorescence in a sample rabbit's blood during the progression of atherosclerosis. 

Figure 3 shows that the maximum fluorescence peak wavelength shifted towards the visible region for HCD group, and it returned towards the control maximum fluorescence peak wavelength for rabbits fed on HCD + Zn. The fluorescence emission from normal rabbit blood serum was significantly different from those for HCD and HCD + Zn. 

One of the first attempts to use laser spectroscopy for plaque diagnosis was made by Kittrell [12], in which an excitation wavelength of 480 nm was used to induce autofluorescence in aorta affected with atherosclerosis. They demonstrated that fluorescence emission from normal artery was significantly different from fibrous plaque. The research was focused on autofluorescence emission for tissue characterization and for use as laser angioplasty guidance system. However, due to the poor biochemical information carried out by fluorescence emission spectrum, the use of Raman spectroscopy for biological tissue classification has been evidenced [17–19].

**Figure 4:**
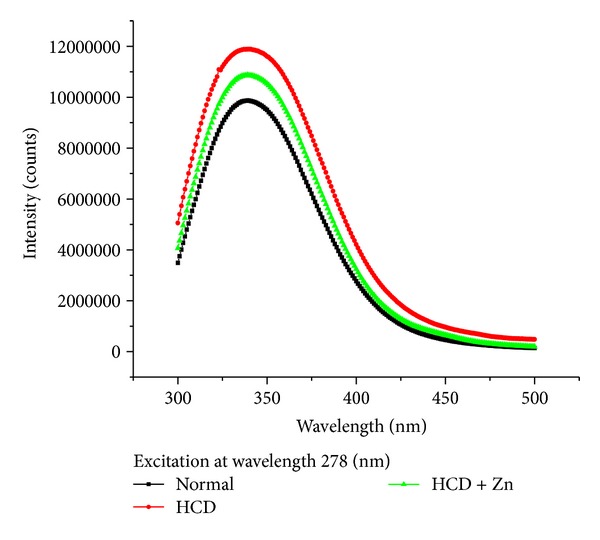
Fluorescence intensity (counts) against wavelength (nm) for a sample of rabbit's blood serum taken from control, HCD, and HCD + Zn groups at excitation wavelength of 278 nm.

Figure 4 shows fluorescence intensity (counts) against wavelength (nm) for a sample of rabbit's blood serum taken from control, HCD, and HCD + Zn groups. In which an excitation wavelength of 278 nm is used to induce autofluorescence in a sample rabbit's blood during the progression of atherosclerosis. The maximum fluorescence peak wavelength returned towards the control maximum fluorescence peak wavelength for rabbits fed on HCD + Zn. The fluorescence emission from normal rabbit blood serum was significantly different from those for HCD and HCD + Zn. 

In this study, we have observed the same results in each group (*N* = 5), so we have provided the average fluorescent peak value of each group (*N* = 5) in terms of mean ± SD, and the statistical analysis between control, HCD, and HCD + Zn was statistically significant.

The best spectroscopic results were obtained with an excitation wavelength of 325 nm. In samples with severe atherosclerotic lesions, the fluorescence spectra showed a significant reduction of the emitted wavelength intensities when compared to normal tissue. There was a clear separation of the fluorescence spectra between normal and mild as well as between normal and severe atherosclerotic lesions; normal tissue showed an increased intensity in the range from 420 nm to 540 nm, whereas atherosclerotic lesions had no or only a small peak at 480 nm. 

Silveira et al. [22] have taken comparable result, where 111 fragments of coronary arteries were analyzed by rear-infrared Raman spectroscopy using 95 samples correctly classified, with 87% agreement and high sensitivity and specificity values. It has shown that FT-Raman can accurately discriminate biochemical differences between normal and atherosclerotic plaque and can be used to spectrally classify them. These results suggest that FT-Raman spectroscopy may be turned to a promising diagnostic technique in therapeutics such as laser angioplasty and as a Raman guidance system. FT-Raman spectrum not only discriminates between healthy and pathological artery wall with molecular specificity, but also provides exclusive diagnostic signature for each tissue. This technique will also enable the study of atherogenesis in situ, allowing the investigation of the disease progression as well as the response of different therapeutic modalities.

Proteins are dynamic and essential to their function. A decrease in absorbance at 280 nm indicates abnormal motion and reflects a deviation from normal structure and function. The extent of deviation reflects the degree of globin unfolding and random motion of Hb molecule under different levels of oxidative stress [23]. Hypercholesterolemia can increase the cholesterol content of platelets, polymorphonuclear leukocytes, and endothelial cells so that endothelial and smooth muscle cells, neutrophils, and platelets may be sources of oxygen-free radicals [24]. It becomes evident that oxidative stress occurs when there is an excessive production of free radicals in the face of defective antioxidant defenses.

### 3.3. Histological Analysis of Rabbit's Aorta

**Figure 5:**
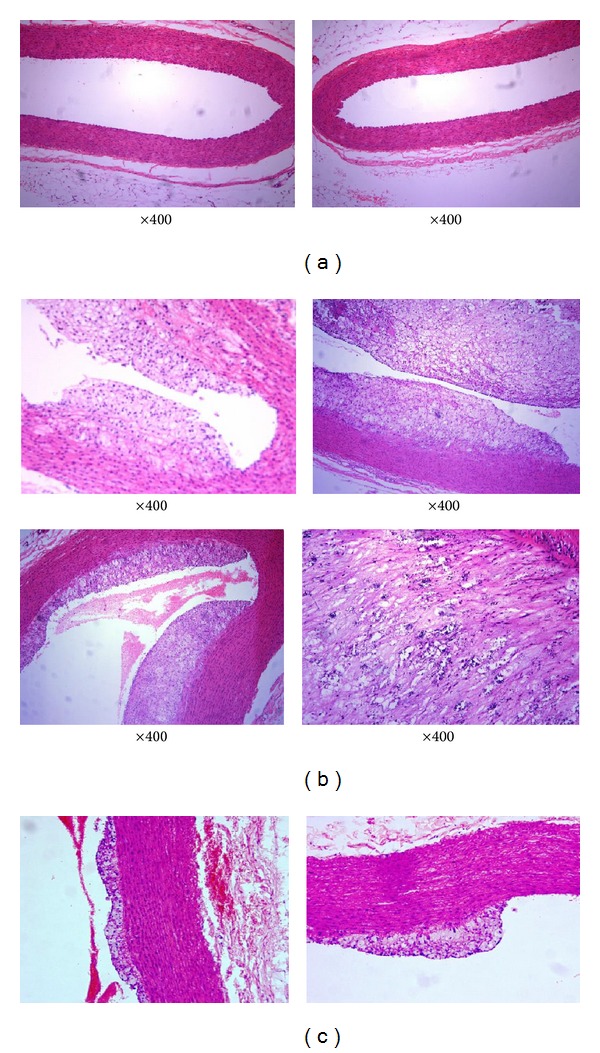
(a) Shows normal aorta. (b) Shows high-density cholesterol aorta. (c) Shows high-density cholesterol + zinc aorta.

Figure 5 ((a) Normal Aorta) demonstrates dilated, benign-looking aorta. Aortic section of rabbit showed well-formed dilated lumen lined by endothelial cells surrounded by their muscular wall. No pathological feature was detected.

Figure 5 ((b) High-density cholesterol Aorta) demonstrates aortic sections of rabbit aorta and showed dilated lumen with different grades of vascular occlusion of the lumen by arthrosclerosis with subintimal deposition of fibrofatty tissue associated with smooth muscle proliferation ranging from partial to subtotal closure of lumen with occasional foci of calcifications.

Figure 5 ((c) high-density cholesterol + Zinc aorta) demonstrates sections of aorta rabbits and showed variable degree of elevation and partial closure of aortic lumen due to atherosclerosis with some differentiation between aortic sections with HCD. 

### 3.4. Biochemical Analysis of Rabbit's Blood Serum

Table 1 shows the levels of TC, TG, and HDL concentrations in control, HCD, and HCD + Zn rabbits. Table 1 indicates significant increases in TC, TG, and HDL in HCD and HCD + Zn compared with control, while significant decreases in TC, TG, and HDL in HCD + Zn compared with HCD.

Table 2 shows that RBCs count, hemoglobin, HCT%, monocyte%, neutrophils%, eosinophils%, and basophiles%, decreased in HCD compared with the control, while WBCs count, LYM%, MCV, MCH, MCHC, RDW, PLT, MPV, PCT, and RDW increased in HCD compared with control. The zinc supplementation to HCD improved all blood indices during the progression of atherosclerosis. The deficiency in RBCs, HGB, and HCT% are very important factors during the progression of atherosclerosis. The dimensional parameters such as RDW, MPV, PCT, and PDW, and PLT count can also be used as predictor factors for atherosclerosis and cardiovascular diseases.

This study suggests that further experiments will be performed taking into consideration lipid peroxidation assay (MDA or TBARS) to show the degree of oxidation in rabbit serum, which will support and link the UV-Visible, fluorescence spectroscopy data, and histopathological data in each group. In addition, we will examine the degree of atherosclerosis in the aorta by measuring the intimal and media thickness of the aorta and expressing it in terms of intimal/media ratio. Finally, we will perform UV-Visible and fluorescence spectroscopy of rabbit serum at different time points (for example 4, 8, and 12 weeks) instead of only one time point (12 weeks), after feeding with HCD to monitor whether the UV-Vis/fluorescent spectroscopy data correlate with the progression of atherosclerosis. 

## 4. Conclusions

To diagnose or detect and delay the progression of atherosclerosis, noninvasive techniques such as ultraviolet-visible and fluorescence spectroscopy techniques were applied on blood sample of rabbits fed on HCD and HCD supplemented with high Zn level for a feeding period of 12 weeks.

Hypercholesterolemia can increase the cholesterol content of platelets, polymorphonuclear leukocytes, and endothelial cells so that endothelial and smooth muscle cells, neutrophils and platelets may be sources of oxygen-free radicals. It becomes evident that oxidative stress occurs when there is an excessive production of free radicals in the face of defective antioxidant defenses. 

At excitation of 192 nm, the fluorescence peak shifted towards the visible region for HCD group, while it returned towards the control fluorescence peak for rabbits fed on HCD + Zn. The fluorescence emission from normal rabbit blood serum was significantly different from those for HCD and HCD + Zn. 

At excitation of 278 nm, the fluorescence peak for normal rabbit blood serum was significantly different from those for HCD and HCD + Zn.

The absorbance peak for rabbits fed on HCD significantly increased compared with the control, while it returned towards the control absorbance peak for rabbits fed on HCD + Zn. The absorbance peak for control was significantly different from those for HCD and HCD + Zn. 

Proteins are dynamic and essential to their function. An increase in absorbance at 280 nm might indicate abnormal protein motion and reflects a deviation from the normal structure and function. The extent of deviation reflects the degree of globin unfolding and random motion of Hb molecule under different levels of oxidative stress.

This study demonstrates that ultraviolet-visible and fluorescence spectroscopy techniques can be applied as noninvasive techniques on a sample blood serum for diagnosing or detecting the progression of atherosclerosis. The Zn supplementation to rabbits fed on HCD delays or retards the progression of atherosclerosis. Inducing anemia in rabbits fed on HCD delays the progression of atherosclerosis. These conclusions might be supported by further histological investigations in rabbit's different organs.

## Figures and Tables

**Table 1:** Concentrations of lipids and lipoproteins in control, HCD, and HCD + Zn Rabbits (*means *P* < 0.05).

Chemical parameters (mg/dL)	Control (*n* = 5)	HCD (*n* = 5)	HCD + Zn (*n* = 5)
Total cholesterol (TC)	65.2 ± 8.71	658.95 ± 95.27*	93.12 ± 5.5*
Low-density lipoprotein (LDL)	43.6 ± 7.41	677.2 ± 11.1*	95 ± 24.1*
High-density lipoprotein (HDL)	12.37 ± 1.65	17.32 ± 1.55	13 ± 1.6
Triglyceride	89.24 ± 9.71	197.25 ± 32.25*	109 ± 10.3*

**Table 2:** Complete blood picture of control, HCD, and HCD + Zn rabbits (*means *P* < 0.05).

Blood index	Control (*n* = 5)	HCD (*n* = 5)	HCD + Zn (*n* = 5)
WBC (K/UL) count	6.95 ± 0.44	13.9 ± 0.57	8 ± 0.52
LYM%	43.73 ± 2.02	60 ± 2.00*	50 ± 2.00
MON%	2.23 ± 1.27	1.15 ± 0.15*	2.26 ± 0.97
NEU%	46.55 ± 2.69	35.7 ± 2.10*	43.21 ± 2.33
EOS%	2.0 ± 0.57	1.33 ± 0.66*	1.6 ± 0.50
BAS%	0.50 ± 0.18	0.45 ± 0.05	1 ± 0.13
RBCs (K/UL) count	6.10 ± 0.14	4.23 ± 0.48*	5.64 ± 0.50
HGB (g/dL)	12.53 ± 0.28	10.56 ± 0.31*	12.47 ± 0.83
HCT%	40.41 ± 0.81	32.42 ± 2.05*	34.34 ± 5.19
MCV	66.27 ± 0.77	79.62 ± 6.75*	70.56 ± 6.20
MCH	20.43 ± 0.40	26.8 ± 4.14	28.62 ± 6.36
MCHC	31.01 ± 0.15	32.92 ± 1.98	33.98 ± 2.56
RDW	13.2 ± 0.34	16.36 ± 1.09*	14.42 ± 1.87
PLT	398.83 ± 50.00	328.87 ± 52*	125.35 ± 12.45
MPV	6.6 ± 0.21	8.7 ± 0.44*	6.1 ± 0.29
PCT	0.25 ± 0.03	0.35 ± 0.06*	0.22 ± 0.05
PDW	9.97 ± 0.55	11.1 ± 0.76*	9.23 ± 0.82
